# Incidence of Paradoxical Neurosensory Detachment in Diabetic Eyes Undergoing Hemodialysis for End-Stage Renal Disease

**DOI:** 10.7759/cureus.14739

**Published:** 2021-04-28

**Authors:** Kshitiz Kumar, Santosh Balasubramaniam, Pallavi Raj, Amar Agarwal

**Affiliations:** 1 Vitreo-Retina, Disha Eye Hospital, Kolkata, IND; 2 Vitreo-Retina, Dr. Agarwal's Eye Hospital (Kolkata Branch at Peerless Hospital & B K Roy Research Centre), Kolkata, IND; 3 Anterior Segment & Glaucoma, Sankar Nethralaya, Kolkata, IND; 4 Anterior Segment & Cataract, Dr. Agarwal's Eye Hospital (Kolkata Branch at Peerless Hospital & B K Roy Research Centre), Kolkata, IND

**Keywords:** hemodialysis, diabetic macular edema, paradoxical neurosensory detachment, dexamethasone implant, central macular thickness

## Abstract

Introduction

Ocular fluid dynamics are known to improve during hemodialysis, and the improvement of uremia after dialysis may lead to osmotic pressure changes in the retina, which eventually affect retinal edema. Recent studies using optical coherence tomography (OCT) to assess the effect of hemodialysis on macular thickness have shown variable results with a majority of them finding a decrease in retinal thickness. Paradoxical neurosensory retinal detachment (NSD) may be defined as the accumulation of subretinal fluid under the macula in patients who are on continuous HD. The purpose of the study was to find out the incidence of paradoxical neurosensory detachment in diabetic eyes undergoing hemodialysis (HD) and its management.

Methods

This was a cross-sectional, prospective study involving end-stage renal disease (ESRD) patients secondary to diabetes. This study evaluated the changes in macular thickness in diabetic retinopathy patients with and without diabetic macular edema (DME) by spectral-domain optical coherence tomography (SD-OCT) 60 minutes before and after HD for ESRD.

Results

Sixty-three eyes (36 patients) were included, with a mean age of 58.2±9.8 years. Seven eyes had paradoxical NSD at presentation with an incidence of 11.11%. Eyes with DME (Group A) showed a significant reduction in central macular thickness (CMT) by 28±2μm post HD, compared to eyes without DME (Group B) where CMT decreased by 15±2μm (p=0.003). Massive subretinal fluid accumulation (paradoxical NSD) with mean CMT 675.57±69.41μm recovered to 250.71±46.79μm at the final follow-up. Five eyes underwent an intravitreal dexamethasone implant (DEX-I, Ozurdex; Allergan, Dublin, Ireland) to achieve the resolution of SRF, whereas two eyes improved spontaneously by nine months.

Conclusion

Hemodialysis results in a decrease of macular thickness in diabetic eyes with or without DME. Paradoxical neurosensory detachment can develop in eyes of patients undergoing HD chronically. Intravitreal dexamethasone implant (DEX-I, Ozurdex; Allergan, Dublin, Ireland) results in early amelioration of such a complication.

## Introduction

Chronic renal failure (CRF) is a condition where there is a loss of kidney function over a period of months or years. There are five stages of CRF based on the glomerular filtration rate (GFR), and dialysis is preferred in stage 5 (GFR<15 ml/min/1.73 m^2^); this stage is also called end-stage renal disease (ESRD) [1]. Dialysis, the process of removal of waste and extra water from the blood, is performed for CRF patients with ESRD. ESRD patients who are on hemodialysis (HD) chronically can develop a wide range of ophthalmologic pathologies, namely, refractive errors, dry eyes, increased tear osmolarity, conjunctival calcium deposits, band keratopathy, corneal endothelium changes, and lenticular opacities. Ocular posterior segment changes, such as retinopathies secondary to diabetes mellitus (DM), hypertension, anemia, and uremia, are also observed frequently in ESRD patients [2].

Diabetic macular edema (DME) is the most common cause of visual loss in diabetic retinopathy (DR), affecting approximately 6.8% of people with diabetes and nearly 20% of patients with DR [3]. During HD, osmotically active materials are eliminated by ultrafiltration, resulting in blood volume depletion, an increase in the plasma protein concentration, and a decrease in serum osmolarity, which, in turn, leads to vascular refilling from the interstitial and intracellular space [4]. Effects of HD on retinal thickness and DME have been evaluated in only a few studies, with conflicting results. Fluorescein angiography-based studies have shown a resolution of hard exudates and a decrease in macular edema in some [5-6] while another study stated that HD did not reduce diabetic macular leakage [7]. Optical coherence tomography (OCT)-based evaluation has also shown varied results, with a trend of decrease in retinal thickness following HD found in some studies [8-9], in contrast to no change in foveal thickness following HD as seen in other studies [10-11].

OCT-based classification of DME has characterized the morphological features of DME into five different patterns: diffuse retinal thickening (DRT), cystoid macular edema (CME), neurosensory retinal detachment (NSD) without posterior hyaloidal traction, posterior hyaloidal traction (PHT) without tractional retinal detachment (TRD), and PHT with TRD [12-13]. NSD under the fovea has been reported in 3%-31% of patients with DME [14-15]. The presence of NSD is found to adversely affect the prognosis of DME.

With the expectation of a decrease in macular thickness following HD, fresh accumulation of subretinal fluid resulting in neurosensory detachment in patients who are already on HD can be termed “paradoxical accumulation.” Despite there being a loss of body fluids, fresh NSD in diabetic patients undergoing HD is intriguing and has never been described before.

Therefore, to find out the incidence of ‘paradoxical NSD’ in eyes undergoing HD, this paper evaluated the changes in macular thickness in diabetic retinopathy patients with and without DME by OCT before and after HD for ESRD. This paper also discusses briefly the management of such an event.

## Materials and methods

This was a cross-sectional study conducted on patients with ESRD secondary to diabetes mellitus who were undergoing hemodialysis (HD) in a tertiary care multi-specialty hospital in Eastern India from March 2016 to January 2020. The study was approved by the Institutional Review Board and adhered to the tenets of the Declaration of Helsinki.

To find out the incidence of paradoxical NSD in diabetic retinopathy-affected eyes in patients who were undergoing hemodialysis thrice a week for at least four hours per session, the components of the following protocol used was based on the study by Jung et al. [16] and Azem et al. [17].

Inclusion criteria

1. Presence of DR with or without DME

2. Visual acuity to an extent that enabled co-operation for a detailed examination

3. Spectral domain-optical coherence tomography (SD-OCT) images of good quality (a signal strength equal to or above 6) 

Exclusion criteria were the presence of non-diabetic retinopathy or media opacities (cataract, vitreous hemorrhage) precluding good OCT examination and systemic or cognitive conditions interfering with OCT examination.

Consecutive patients who fulfilled the above criteria and were willing to participate in the study were enrolled. All patients underwent a detailed ophthalmologic examination, including best-corrected visual acuity on Snellen’s Chart (BCVA), slit-lamp examination, including bio-microscopy before the HD session. Dilated SD-OCT (Cirrus HD-OCT; Carl Zeiss Meditec, Dublin, CA) was performed 60 minutes before and 60 minutes after an HD session on all patients at the same time intervals. Based on the 6 mm x 6 mm data cube captured by the Macular Cube 512x128 scan protocol, using the Early Treatment Diabetic Retinopathy Study (ETDRS) grid map cantered on the fovea, central subfield/macular thickness (CMT) from the internal limiting membrane (ILM) to the retinal pigment epithelium (RPE) in microns (μm) was noted for each eye before and after HD. Patients were divided into two groups based on the presence or absence of DME: Group A with macular edema (increased macular thickness in biomicroscopy and the presence of DME on OCT) and Group B without macular edema.

Intraocular pressure (IOP) was measured using the Goldmann Applanation Tonometer (GAT) before and after HD. In addition, body weight and blood pressure were measured before and after each HD session to calculate the change in body weight and mean arterial blood pressure. The mean arterial blood pressure (MABP, mmHg) was measured as diastolic blood pressure + ⅓ (systolic blood pressure - diastolic blood pressure). The change in body weight represented the amount of fluid removed during HD [16]. HbA1C and serum albumin levels were also noted from the medical record sheet of the HD unit.

Statistical analysis

Statistical analysis was performed with the Statistical Package for the Social Sciences (SPSS) software version 22.0 (IBM Corp, Armonk, NY). Quantitative variables were described through the mean and standard deviation. We included both eyes of a patient for analysis. The level of statistical significance was taken into account if p < 0.05. CMT, IOP, MABP, and body weight before and after hemodialysis were compared by the paired 𝑡-test within the DME (Group A) and non-DME (Group B) groups. The Mann-Whitney U test was used for a comparison of continuous data between the DME (Group A) and non-DME (Group B) subgroups. Pearson’s correlation test was used to investigate possible correlations between changes in CMT and mean blood pressure (BP), body weight, glycated hemoglobin (HbA1C), and albumin levels.

## Results

Sixty-three eyes of 36 patients (20 males, 16 females) with a mean age of 58.2±9.8 years were enrolled in the study. Eight eyes of these patients were excluded for not meeting the inclusion criteria. One patient had an eye enucleated.

The mean age, duration of diabetes, and duration of HD treatment were significantly different between those eyes with DME (Group A) and those without DME (Group B) along with the HbA1C level (p<0.05; Mann-Whitney test). MABP before and after HD was significantly lower in Group A (with DME) as compared to Group B (without DME) [p<0.05 Mann-Whitney test]; however, the change in MABP in each group was not significantly different (p>0.05). Weight loss post-dialysis session was similar in both groups (p>0.05) (Table 1).

**Table 1:** Clinical and laboratory data of groups with and without DME undergoing HD HbA1C: glycated hemoglobin; DME: diabetic macular edema; HD: hemodialysis

Parameters	Group A (with DME) {n=31eyes}	Group B (without DME) {n=32eyes}	p-value
Age (years)	63.8 ± 8.2	55.4 ± 12.1	0.003
Duration of Diabetes	14.50 ± 6.32	12.67 ± 8	0.016
Duration of HD (years)	4.33 ± 1.5	6.33 ± 2.3	0.034
HbA1C (%)	7.6 ± 1.2	6.21 ± 1.4	0.04
Albumin (gram)	36.57 ± 2.33	35.51 ± 2.14	0.397
s-BP (mmHg) before HD	133.2 ± 20.6	141.9 ± 26.3	
d-BP (mmHg) before HD	74.6 ± 8.8	85.8 ± 16.9	
s-BP (mmHg) after HD	126.9 ± 28.1	133.6 ± 36.9	
d-BP (mmHg) after HD	82.8 ±1 4.7	86.1± 14.2	
Mean BP before HD	93.66 ± 14.00	103.66 ± 18.03	0.03
Mean BP after HD	92 ± 10.12	101.66 ± 8.05	0.026
Mean BP difference before and after HD	-1.66 ± 3.88	-2 ± 9.98	0.612
Weight loss (kg)	-2.7 ± 1.4	-2.8 ± 1.2	0.120

**Figure 1:**
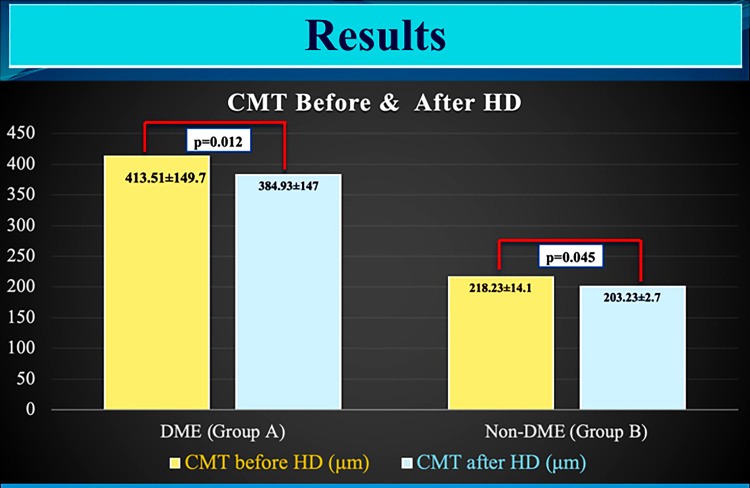
Bar graph showing the intragroup central macular thickness (microns) pre- (yellow color) and post- (light blue) hemodialysis with p-values (paired t-test)

The average CMT of Group A eyes with DME decreased from 413.51±149.73 to 384.93±147.04 μm (p=0.012; paired t-test), whereas the average CMT of Group B eyes with non-DME decreased from 218.23±14.1 to 203.2±32.7 μm after HD (p=0.045; paired t-test). Eyes with DME (Group A) showed a trend toward reduction in CMT by 28±2 μm post HD, whereas in eyes without DME (Group B), CMT decreased by 15±2 μm after HD and this trend was statistically significant between the groups (p=0.003; Mann-Whitney test) (Figure 1).

The mean IOP decreased from 15.1±2.8 to 13.9±2.2 mmHg after HD in Group A and decreased from 14.1±2.3 to 13.0±1.5 mmHg in Group B. The decline in IOP was not statistically significant (p=0.539) between the groups.

No significant correlations were observed between the change in CMT thickness and the serum albumin level (r=-0.324, p=0.627), HbA1C (r=0.361,p=0.282), body weight (r=0.462,P=0.124), and MABP (r=-0.513,p=0.467). Also there was no correlation between change in IOP and that of body weight (r=0.472, p=0.517).

Paradoxical response

**Figure 2:**
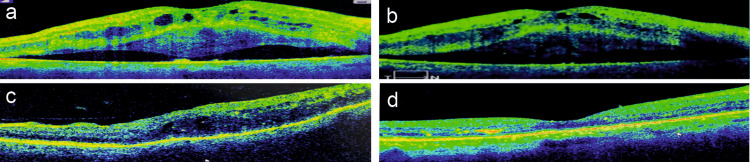
OCT images of Patient 1 Pre- and post-intravitreal Ozurdex implant of the right eye (a,c) and left eye (b,d) at one month showing the resolution of paradoxical neurosensory detachment OCT: optical coherence tomography

Despite being on hemodialysis for a mean duration of 4.33±1.5 years, seven eyes of four patients of Group A presented with an accumulation of SRF resulting in neurosensory detachment underneath macula post-hemodialysis session within 24 hours. We labeled this atypical finding at presentation a ‘paradoxical response.’ The incidence of paradoxical NSD in this study was 11.11% (7/63). The DRT type of DME was present in four eyes and CME type in three eyes for which they have been treated before. No specific OCT attribute could be found to predict the development of paradoxical NSD in the subgroup A analysis. After one month of continuous bi-weekly hemodialysis sessions with no resolution in NSD, an intravitreal dexamethasone implant (DEX-I, Ozurdex; Allergan, Dublin, Ireland) was injected in five eyes of three patients. After four weeks of implant injection, NSD resolved completely without recurrence till the fourth month of the follow-up period (Figure 2).

**Figure 3:**
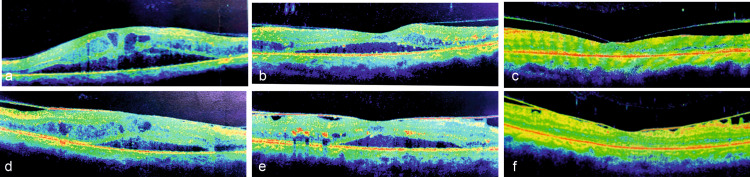
OCT images of Patient 4 (a&d) Right eye and left eye at presentation showing macular edema with neurosensory detachment, (b&e) at four months showing the beginning of the resolution of subretinal fluid and edema, and (c&f) at the ninth month showing completely resolved paradoxical NSD on bi-weekly hemodialysis in the respective eyes OCT: optical coherence tomography; NSD: neurosensory retinal detachment

In the remaining two eyes of a patient who could not afford implant injection, NSD persisted for the initial four months before it started spontaneously resolving with bi-weekly HD and by the ninth month of presentation, NSD had resolved completely with good anatomical and functional recovery (Figure 3).

The mean CMT in this subset of patients improved from 675.57±69.41 μm to 250.71±46.79 μm at the final visit. Table 2 summarizes the vision and OCT characteristics of all the above eyes.

**Table 2:** Visual acuity and macular thickness pre- and post-treatment for paradoxical neurosensory detachment * Patients receiving intravitreal Ozurdex implant for a paradoxical response, recovery period at four weeks; ** Patient who did not receive an implant in any of the eyes, recovery period of nine months on bi-weekly hemodialysis

Patient	Eye	BCVA (Snellens)		Central Macular Thickness (mm)	
		Presentation	Post- Recovery	Presentation	Post – recovery
1^*^	OD	3/60	6/36	730	290
	OS	6/60	6/9	760	218
2*	OD	6/60	6/36	680	170
	OS	4/60	6/60	546	312
3*	OD	6/60	6/18	690	265
4**	OD	6/24	6/18	688	245
	OS	6/36	6/18	635	255

## Discussion

Advanced chronic kidney disease (CKD) or end-stage renal disease (ESRD) patients suffer from volume overload and uremia. The removal of fluid during dialysis treatment, also known as ultrafiltration, is the cornerstone of volume management in ESRD [18].

Ocular fluid dynamics are known to improve during hemodialysis and the improvement of uremia after dialysis may lead to osmotic pressure changes in the retina, which eventually affect retinal edema [8,16].

Recent studies using OCT to assess the effect of hemodialysis on macular thickness have shown variable results with a majority of them finding a decrease in thickness. This study included subjects having diabetic retinopathy and subgroups with DME so that the incidence of paradoxical NSD could be evaluated in this cohort. In this study, both in the DME group as well as in the non-DME groups, there was a significant decrease found in CMT post-HD. Although the average amount of decrease was more in the group with DME (28 μm vs 15 μm), the study did not evaluate the corresponding clinical/functional improvement. Pahor et al. reported a significant reduction in retinal thickness among patients who required hemodialysis for different reasons, compared with healthy controls but in their study, subjects underwent a single OCT exam at time points independent of the hemodialysis session [19]. In this study, however, OCT examination was performed 60 minutes pre and post-HD so that sufficient time was there for patient mobilization given their comorbidities and accounting for the distance to the dialysis department in a different wing of the hospital. It is recommended to perform OCT 30 minutes before and after HD, as patients who noted ocular side effects, such as blurred vision or eye pain, experienced them soon after the treatment, possibly due to the massive changes in osmolality caused by the hemodialysis [19-20]. However, for the above reasons, we did it at a 60-minute interval.

Similar to our study, Theodossiadis et al. detected a significant mean decrease in maximum macular thickness in eyes with DME by 31 μm after each session of HD as well as in the average macular thickness by 10 μm with a less pronounced effect found in the non-DME group [8]. Jung et al. in their study showed a similar trend of average decrease in central subfield thickness of around 7 μm with a marked decrease of 15 μm in the DME group as compared to only a 1.5 μm decrease in the non-DME group [16]. Our study results concurred with the above findings.

In contrast to the results discussed so far, Auynet et al. in their study on diabetic eyes without DME reported no change in foveal thickness after one session of HD apart from a slight (2%) and non-significant reduction [10]. Similarly, Emre et al. did not find a significant change in foveal thickness 30 minutes post-HD in patients of chronic renal failure [11].

The above studies had a significant limitation of evaluating change in macular thickness before and after a single session of HD. The long-term effect of HD on the macular thickness and DME has not been studied extensively. A fundus fluorescein angiography (FFA)-based study by Tokuyama et al. showed that macular leakage was unchanged in 70% of eyes at four weeks as compared with baseline appearance and concluded that hemodialysis does not benefit macular leakage in diabetic patients receiving hemodialysis for ESRD [7]. Matsuo et al. reported the resolution of hard exudates in two eyes six months after the start of hemodialysis [5]. Jung et al. mentioned that in three subjects with clinically significant macular edema, the size of intraretinal cystic lesions decreased and intraretinal fluid and retinal thickness decreased further after a single HD session [16]. More recently, Hwang et al. in their study on the effect of initiation of HD for the first time in CRF patients found that not only macular thickness decreased significantly by 30 microns on SD-OCT but also the incidence of any DME decreased from 69% to 26% with concomitant improvement in DME at one month [9].

From the evidence discussed so far and the positive trend of decrease in central macular thickness post one session of HD in our study sample, an 11.11% incidence of huge NSD under the macula at presentation despite patients being on HD for an average of four years in the DME group makes this a unique clinical finding. We label this unreported entity as ‘Paradoxical Neurosensory Detachment’ in DME patients undergoing HD. The underlying mechanism behind this entity could be explained based on two proposed theories - Oxidative Stress Theory & Dialysis Disequilibrium Syndrome.

Oxidative stress theory for paradoxical NSD

The main cause of CRF is a damaged kidney. Ongoing inflammation is the main reason for the diseased kidney, which does not respond to medications. Vedakedath et al. mentioned reiterated that in patients undergoing dialysis, there is an increased probability that the chronic inflammation of the kidney is accelerated, leading to further complications [21]. This inflammation, in turn, plays an important role in the development of oxidative stress in patients undergoing dialysis. In the paper, they enumerated the following mechanism leading to the build-up of oxidative stress during chronic hemodialysis: the membrane of dialysis gets subjected to an immunological response by low molecular weight substances that include immunoglobulin G (IgG), the complement component, and thereby making this membrane permeable to granulocytes. The activated granulocytes in the blood stimulate the release of reactive oxygen species (ROS) and exaggerate oxidative stress [21]. It was also found that there are reduced trace elements, such as copper and zinc, and superoxide dismutase (SOD) levels among post-dialytic persons [22]. Also, the non-functioning kidney activates macrophages, vascular cells, and various glomerular cells to produce free radicals, which further aggravates the oxidative stress [21]. Other papers stated that during dialysis, there is a possibility of the retention of inflammatory markers, the development of oxidative imbalance, and the activation of the complement [23]. This state of persistent oxidative stress leads to altered endothelial function thereby resulting in increased vascular permeability which, in turn, causes the accumulation of water in interstitial spaces and edema. 

NSD or subretinal detachment form of DME has been attributed before to the disruption of tight junctions and the breakdown of the retinal pigment epithelium (RPE) pump that may result in fluid accumulation, which exceeds the absorption capacity of the RPE pump for subretinal fluid [24].

Some studies showed that inflammation plays a crucial role in NSD in DME. In a study evaluating the different morphological macular edema types, the levels of inflammatory factors in the aqueous humor - including interleukin 6 (IL-6), IL-8, IP-10, and platelet-derived growth factor-AA - were higher in the NSD group than in the diffuse retinal thickening (DRT) group [24]. Sonoda et al. reported that intravitreal IL-6 is the factor that was most significantly associated with NSD [25]. Moreover, Kaya et al. demonstrated that the serum chitinase-3-like protein 1 and IL-6 levels were significantly higher in DME with NSD than in patients with DRT and CME [26].

Therefore, in patients who have been on dialysis for a long time, there is a risk of accelerated edema and subretinal fluid accumulation because of oxidative stress-related inflammation resulting in huge neurosensory detachment as was seen in this series.

Dialysis disequilibrium syndrome theory for paradoxical NSD

The dialysis disequilibrium syndrome is defined as a clinical syndrome of neurological deterioration that is seen in patients who undergo hemodialysis [27].

The basic underlying pathology is the development of cerebral edema as a consequence of the dialysis procedure. It is more likely to occur in patients during or immediately after their first treatment but can occur in any patient who receives hemodialysis. The principal factor leading to the disequilibrium syndrome is the formation of an osmotic gradient causing water to move into the brain resulting in cerebral edema. The theory behind the osmotic gradient is the development of the “reverse urea effect” in which the urea concentration in the CNS remains elevated because of its slower diffusion from the central nervous system (CNS) to the blood than the diffusion of urea from the blood into the dialysate compartment [27].

Chen et al., in their study of hemodialysis patients who underwent diffusion-weighted MRI after their dialysis treatment, found evidence of interstitial cerebral edema and not intracellular edema [28]. Slow removal of urea during the first several treatments is critical for avoiding this syndrome [27].

Based on the possibility of developing cerebral edema in patients on hemodialysis secondary to osmotic gradient as seen in disequilibrium syndrome, the paradoxical accumulation of interstitial fluid in the subretinal space leading to huge NSD can be attributed to the formation of a similar osmotic gradient in the macula. However, the exact nature of such an osmotic gradient in the macula during hemodialysis needs to be evaluated through in vitro and in vivo studies.

From the two theories discussed above, we may propose that patients who are on chronic HD may develop paradoxical NSD due to an oxidative, stress-related, leaky blood-retinal barrier, whereas patients who have been recently put on HD are at risk of this paradoxical NSD if the rate of urea removal is fast as seen in Dialysis Disequilibrium Syndrome. We could not find any specific OCT biomarker to predict the development of paradoxical NSD in eyes with DME. Besides the DME eyes, which developed this exaggerated fluid accumulation underneath, the neurosensory retina had DRT/CME type macular edema. The reason for not finding this paradoxical NSD in eyes without DME could be due to the healthy state of the blood-retinal barrier and the RPE pump as against in the DME group where both the barrier and the pump function is compromised leading to the accumulation of subretinal fluid [24].

In this series of patients with persistent paradoxical NSD at one month, we administered DEX-I for the early amelioration of the subretinal fluid. DEX-I is a biodegradable, slow-release drug delivery system that has been approved for use to treat DME, macular edema secondary to retinal vein occlusion, and non-infectious posterior uveitis. Corticosteroids inhibit retinal VEGF, ICAM-1, and TNF-α expression levels as well as induce the expression of several anti-inflammatory proteins, such as IL-10 and adenosine [29].

DEX-I has been shown to reduce retinal leukostasis. In addition to their anti-inflammatory effects, corticosteroids induce retinal fluid clearance via the transcellular aquaporin-4 and potassium channels in retinal Muller cells [29]. Compared to three loading doses of anti-VEGF agents, aflibercept and ranibizumab, better morphological improvement was observed after one DEX-I injection for DME with NSD [30]. Hence, the choice of injection was DEX-I in this study. One patient with both eyes showing the resolution of paradoxical NSD nine months after presentation points toward the need for early intervention in the form of intravitreal injections, to have quick anatomical and visual recovery.

## Conclusions

This study evaluated the effect of hemodialysis on central macular thickness in diabetic eyes with or without DME and found that in both groups, CMT decreased significantly. Patients who are chronically on hemodialysis are at risk of developing paradoxical neurosensory detachment despite the known beneficial effect of dialysis on DME. The underlying pathogenesis behind such a paradoxical accumulation of subretinal fluid is not clear and further studies are required at the experimental and clinical levels to find out the exact cause. In the event of such an incidence, intravitreal steroids are of proven efficacy for early resolution although spontaneous recovery may happen in the long term with hemodialysis.
